# A novel few shot learning derived architecture for long-term HbA1c prediction

**DOI:** 10.1038/s41598-023-50348-1

**Published:** 2024-01-04

**Authors:** Marwa Qaraqe, Almiqdad Elzein, Samir Belhaouari, Md Shafiq Ilam, Goran Petrovski

**Affiliations:** 1 College of Science and Engineering, Hamad Bin Khalifa University, Qatar Foundation, Doha, Qatar; 2 Sidra Medicine, Doha, Qatar

**Keywords:** Biotechnology, Biomarkers, Endocrinology, Health care, Engineering, Mathematics and computing

## Abstract

Regular monitoring of glycated hemoglobin (HbA1c) levels is important for the proper management of diabetes. Studies demonstrated that lower levels of HbA1c play an essential role in reducing or delaying microvascular difficulties that arise from diabetes. In addition, there is an association between elevated HbA1c levels and the development of diabetes-related comorbidities. The advanced prediction of HbA1c enables patients and physicians to make changes to treatment plans and lifestyle to avoid elevated HbA1c levels, which can consequently lead to irreversible health complications. Despite the impact of such prediction capabilities, no work in the literature or industry has investigated the futuristic prediction of HbA1c using current blood glucose (BG) measurements. For the first time in the literature, this work proposes a novel FSL-derived algorithm for the long-term prediction of clinical HbA1c measures. More importantly, the study specifically targeted the pediatric Type-1 diabetic population, as an early prediction of elevated HbA1c levels could help avert severe life-threatening complications in these young children. Short-term CGM time-series data are processed using both novel image transformation approaches, as well as using conventional signal processing methods. The derived images are then fed into a convolutional neural network (CNN) adapted from a few-shot learning (FSL) model for feature extraction, and all the derived features are fused together. A novel normalized FSL-distance (FSLD) metric is proposed for accurately separating the features of different HbA1c levels. Finally, a K-nearest neighbor (KNN) model with majority voting is implemented for the final classification task. The proposed FSL-derived algorithm provides a prediction accuracy of 93.2%.

## Introduction

Diabetes mellitus (DM), commonly known as diabetes, is a chronic metabolic disorder that has been growing at an alarming rate throughout the world. One of the most accepted biomarkers used for DM diagnosis and management is glycated hemoglobin (HbA1c)^1^. Hemoglobin, a protein in red blood cells, bonds with glucose molecules in the blood. As blood glucose levels increase, more hemoglobin will be coated with glucose molecules. The HbA1c is a blood test that measures the average blood glucose content in the blood over a period of 90–120 days as red blood cells typically live 3 months^2^. The American Diabetes Association (ADA) defines a test value of HbA1c <5.7% as non-diabetic, test values between 5.7% and 6.4% are regarded as pre-diabetes states, while the HbA1c value $\ge$ 6.5% is considered diabetic.

The proper management of diabetes significantly depends on the periodical assessment of HbA1c levels, and thus this test is often performed periodically to classify diabetes severity, indicate the level of management of the disorder, and forecast future complexities^3^. Research has shown that lower levels of HbA1c play an essential role in reducing or delaying microvascular difficulties that arise from diabetes^4–6^. However, there is an association between elevated HbA1c levels and the development of diabetes-related comorbidities. Several studies concluded that HbA1c levels could be used to infer the future progression of diseases such as cardiovascular disease (CVD), nerve, eye, and kidney damage^4^. Specifically, a study among East Asian patients in^7^ found elevated levels of HbA1c to be associated with a higher likelihood of mortality death from CVD. Another study in^8^ correlated HbA1c with mortality and found a resilient connection between elevated HbA1c and mortality among the subjects without a previously known history of diabetes. Diabetic retinopathy is another health complication arising from diabetes and research has shown that a 10% reduction of HbA1c reduces 43% of retinopathy development risk^9^.

Recently, the advent of Artificial Intelligence (AI)-based algorithms and sensor technologies facilitated the creation of techniques aimed at constructing diagnostic and treatment models for diabetes. These models are developed using data obtained from continuous glucose monitoring (CGM) devices and various smart wearables, such as smartwatches, bracelets, and smart clothing^10–14^. The coupling of AI with the CGM data has unlocked important insight into BG variability, prediction of life-threatening events like hypoglycemia and hyperglycemia^11^, blood glucose forecasting^12^, and the determination of optimal insulin dosages to improve the quality of life^13^. Despite the substantial work done in the area of AI and diabetes, there has been no work that has attempted to predict the HbA1c levels of patients, an important factor in diabetes management.

The *prediction* of HbA1c levels of a patient based on their current blood glucose trends can revolutionize the management of diabetes, transforming it from a reactive to proactive approach by allowing patients and physicians to make changes to treatment plans, lifestyle, and diet in the present to avoid elevated HbA1c levels (and their associated consequences) in the future. With the recent advancements in sensor technology, which facilitate the daily monitoring of blood glucose (BG) levels using wearable CGM devices, the prediction of HbA1c is a possibility. These devices provide large sets of data that can be exploited to gain insight into how well a person is controlling their diabetes.

Despite the significance of HbA1c prediction, no work has investigated this idea besides our initial contribution^15^. The work in the literature has focused primarily on *estimating* the current/instantaneous value of HbA1c from plasma glucose values^16–18^. In a clinical study of the Diabetes Control and Complications Trial (DCCT), a correlation was found between HbA1c and mean BG, $\mu _{BG}$^19^. The estimated HbA1c values were compared with the actual HbA1c values and a coefficient of determination ($R^{2}$) score of 0.82 was obtained. Another similar study^20^ known as the A1c Derived Average Glucose (ADAG) also estimated the HbA1c values from $\mu _{BG}$. An $R^{2}$ score of 0.84 was found in the ADAG study. The authors in^21^ investigated the relationship between the HbA1c and $\mu _{BG}$ by using the Pearson correlation and reported an $R^{2}$ score of 0.71. A deep neural network was recently applied to estimate HbA1c among Type 1 Diabetes Mellitus (T1DM) patients^22^. The approach used self-monitoring blood glucose (SMBG) to estimate instantaneous HbA1c and achieved an $R^{2}$ score of 0.71. The work in^23^ implemented a Support Vector Machine (SVM) classifier to detect low and high HbA1c for early diabetes detection and reported an F1 score of 81%. These works have made significant strides in the numerical estimation of instantaneous HbA1c values, a biomarker measured clinically.

Furthermore, it is worth noting that the majority of the methods, with the exceptions of those presented in^11^ and^12^, primarily focus on adult populations in their research. Nevertheless, research conducted by the National Institutes of Health (NIH) and presented at the ADA scientific sessions has highlighted that blood glucose levels measured during childhood examinations can serve as predictors of future diabetes-related complications, including conditions such as eye disease (retinopathy) and kidney disease (nephropathy)^24^. This valuable insight allows us to focus on pediatric Type 1 diabetic children in our study.

However, as highlighted previously, there has been no work dedicated to predicting the value of HbA1c given measurements of current BG, other than^15^, where a model based on ensembling Random Forest (RF) and extreme gradient boosting (XGB) coupled with time range and glucose variability features fusion was developed to predict HbA1c values of pediatric Type 1 diabetic children. The model achieved an $R^{2}$ score of 0.81. A multi-stage multi-class (MSMC) model comprising SVM, Naive Bayes (NB), and RF was also developed for the advanced prediction of HbA1c levels into four distinct categories. This approach achieved an accuracy of 88.65%^15^.

To further the work in this domain, we present a novel approach for HbA1c prediction based on transforming CGM time-series data into images and exploiting an inventive version of a Few-Shot Learning (FSL) model to classify the CGM-based images into HbA1c ranges/classes. Research has shown that Convolutional Neural Networks (CNNs) perform well with images as input and outperform traditional machine learning (ML) techniques^25^. To exploit the benefits of CNNs, this paper presents novel transformations that convert the CGM sensor time-series data into spatial-based images. Additionally, the fundamental challenge with traditional Deep Learning (DL) approaches is that they require large-scale datasets to train DL models. In this study, we implement an FSL method to overcome the data limitation aspect of DL architectures. A novel normalized FSL-Distance (FSLD) metric is also introduced to accurately separate image representations of CGM time series data of patients of different HbA1c classes. Finally, a K-Nearest Neighbor (KNN) model with majority voting was implemented for HbA1c class prediction. The proposed prediction model has boosted the accuracy of HbA1c prediction to $93.2\%$ compared to $88.6\%$ in^15^ when trained with 80% of the data and tested with 20% of the unseen values.

The outcome of the proposed HbA1c prediction system model has the capacity to induce a paradigm shift in the care and management of people living with diabetes. Knowledge of future HbA1c levels enables imminent changes to be made in patients’ treatment plans, if necessary, which can help avoid complications in the future, whereas current approaches necessitate that patients continue with their current treatment plans until the next time HbA1c is checked.

In summary, this work offers the following significant contributions:

1. A novel approach is proposed for missing CGM data estimation for CGM time series.
2. A set of statistical features, derived from CGM data, that are highly correlated with future HbA1c levels are discovered and presented.
3. Two unique time series to spatial image transformations are developed for CGM data.
4. An FSL-based feature extraction method is proposed for CGM-based images, where a novel image distance metric, FSLD, is proposed for separating images corresponding to CGM time series that belong to patients from different HbA1c classes.

The remainder of the paper is outlined as follows: Section Data collection and pre‑processing includes details about data collection and pre-processing. Section Proposed methodology presents the proposed methodology for HbA1c prediction. Section Performance evaluation and discussion discusses the results of our HbA1c prediction model. Section Benchmarking on other datasets/applications benchmarks our FSL-based approach with the state of the art. Finally, Section Conclusion concludes the study, highlighting its major research contributions, and discussing future work.

## Data collection and pre-processing

**Figure 1:**
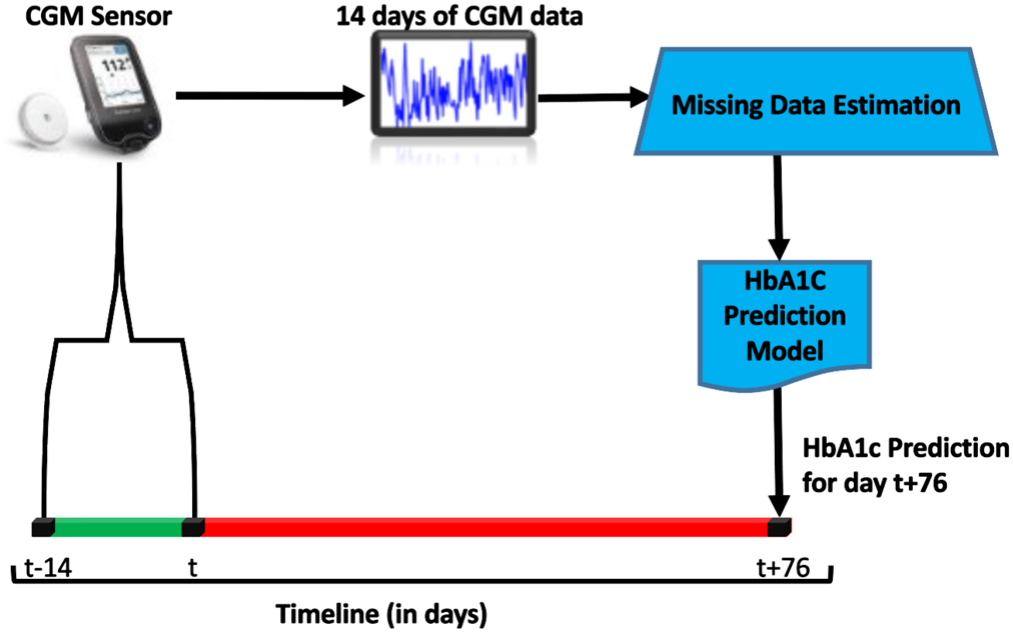
High-level overview of proposed HbA1c prediction methodology.

This section details the data collection effort and the data analysis and pre-processing steps. HbA1c indicates the average BG level over the past two to three months. Thus, to ensure adequate time to make impactful life changes, the target prediction window should be at least two months in advance. As shown in Fig. 1, the proposed algorithm will utilize the past 14 days of a user’s CGM data to predict their HbA1c 76 days in the future. We have adopted a window of 14 days to use in the learning process based on previous research efforts^15^.

### Study population

A retrospective data collection effort was undertaken to collect the BG data from Sidra Medicine, a women’s and children’s hospital as well as a medical education and biomedical research center in Doha, Qatar^26^. Our research plan was approved by the Institutional Review Board (IRB) of Sidra Medicine. All methods were performed in accordance with the guidelines and regulations set in place by Sidra Medicine IRB and by following the policy, regulations, and guidelines for research involving humans set forth by the Ministry of Public Health (MoPH) in Qatar. In addition, informed consent was obtained from all subjects *and* their legal guardians/parents for the use of the relevant data.

All collected data were from pediatric subjects (mean age 12.7 ± 4.5 years; age range 6–22 years) with T1DM who wore the Freestyle Libre CGM sensor as part of their monitoring and management plan.

The CGM sensor consists of a glucose sensor implanted into the body’s subcutaneous tissue. The sensor measures interstitial fluid glucose levels every 15 min, giving 96 measurements per day. The CGM sensors have a lifetime of 14 days, after which they are replaced with new ones. The 14 days of CGM data from the sensor are collected and saved to a secured memory disk. All subjects in the study wore the CGM sensors continuously for 90–120 days. HbA1c levels are clinically measured for each subject on the 90th day of data collection at Sidra Medicine’s laboratory. The collected CGM dataset is summarized in Table 1. The data collection effort utilized the data of two hundred subjects (mean age 12.7 ± 4.5 years; range 6–22 years) with T1DM during the years of 2019 and 2020.

**Table 1:** Summary of CGM dataset.

Total number of subjects	Mean age ± SD (years)	Number of days	CGM device	Samples per day	Mean HbA1c ± SD	HbA1c range (%)
200	12.7 ± 4.5	90–120	FreeStyle Libre	96	8.99 ± 2.13	5.2–14.5

### Data analysis

**Figure 2:**
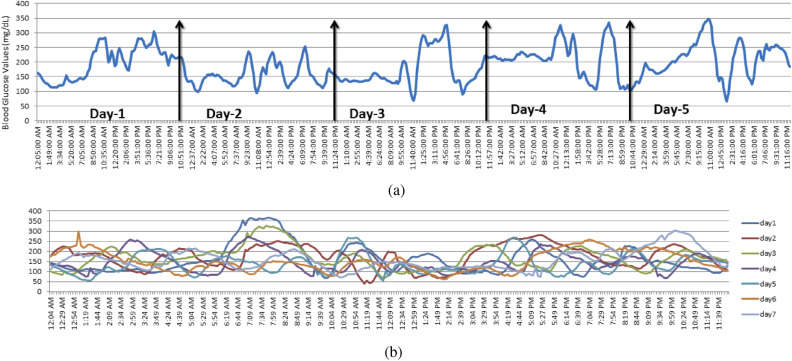
(**a**) Illustration of BG trend in time-series format (**b**) Concatenation of 7 days of CGM data.

Figure 2a depicts a sample of a BG time series for one of the subjects over the course of 5 days. The y-axis represents BG values in mg/dL and the x-axis displays the time stamp for five consecutive days. BG is variable and dependent on many factors, including sleep, type of food eaten, and stress. Figure 2b illustrates the variation in BG trends by concatenating seven 24-h BG readings from different days. As seen from the figure, BG levels rise at different times of the day; however, there were observable peaks that occurred during morning, noon, and evening hours, correlating with typical meal timings. These observations indicate that BG values can *loosely* follow a trend but BG peaks and plummets vary throughout the day. These variations make the data modeling task more challenging and prevent the development of a deterministic model that is capable of explaining BG trends.

### Data preprocessing

#### Missing data estimation

In some instances, a user might take off their CGM device to either replace it after the 14-day period or for other reasons. Additionally, a CGM device might occasionally become dislodged from a user’s arm. Such instances may introduce missing data points in the users’ CGM time series. To address missing data, a pre-processing step is taken to estimate the values of the missing BG readings.

**Figure 3:**
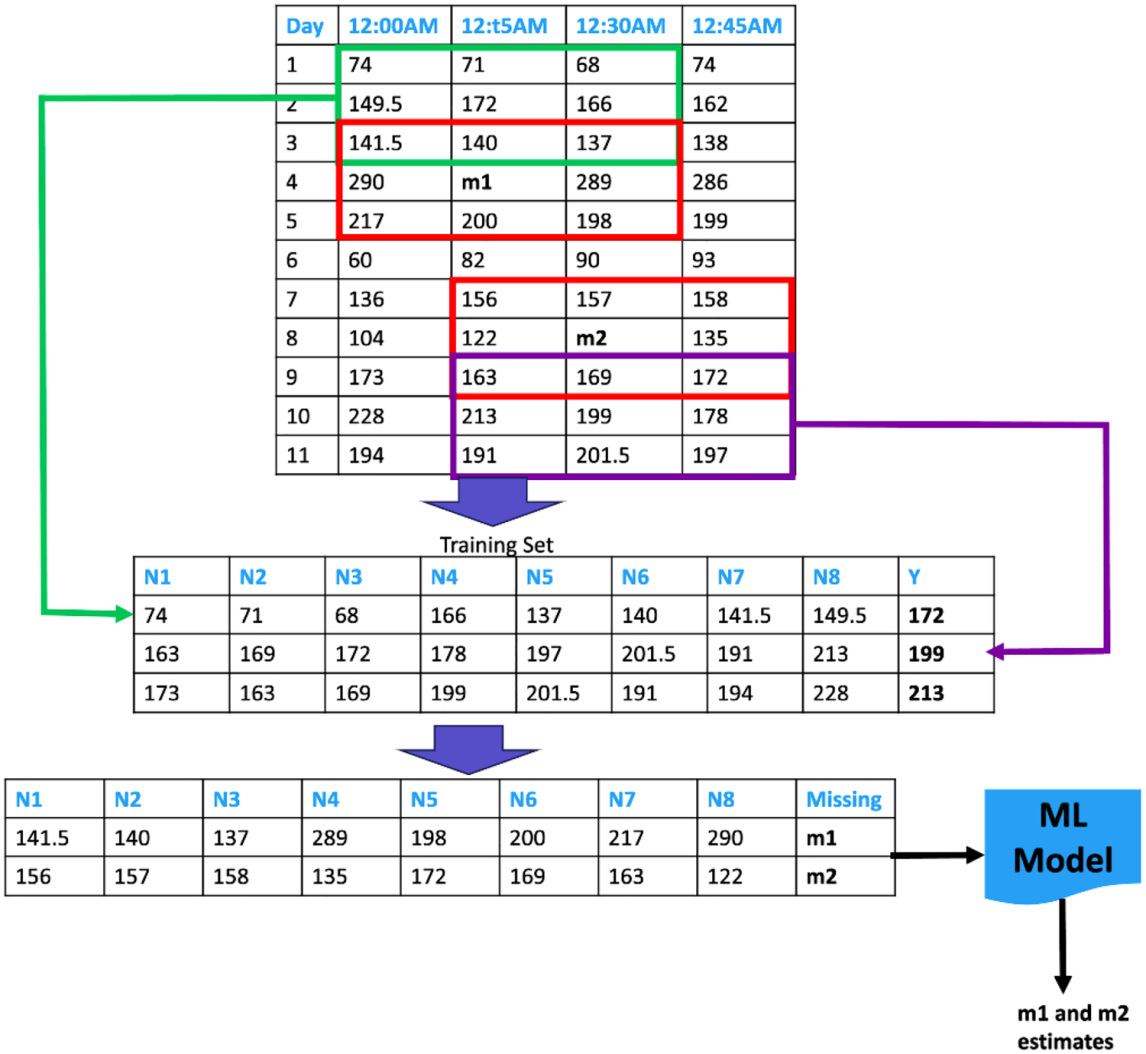
Schematic diagram illustrating the procedure of estimating missing CGM data points using the nearest neighbors method.

To impute missing data points, a Machine Learning (ML) method is adopted. As shown in Fig. 3, a reading in a CGM time series can be defined along two axes, the day of the reading and the time of the reading. This representation reflects the fact that a BG reading is not only correlated with the readings directly before and after it but also readings on different days that are taken at a similar time. This is due to the fact that BG typically follows similar trends, assuming patients follow similar activities and meal timings on most days, which is generally a reasonable assumption. Thus, we hypothesize that a missing BG reading can be accurately estimated by examining readings that take place directly before and after it as well as readings that occur at a similar time frame on adjacent days. For instance, consider the missing BG reading *m*1 in Fig. 3. Note that *m*1 is a reading that should have occurred on day 4 at 12:15 AM. As a result, to estimate *m*1, we consider the readings on day 4 at times 12:00 AM and 12:30 AM. Additionally, we consider readings that occur on days 3 and 5 at times 12:00 AM, 12:15 AM, and 12:30 AM. These readings are the eight adjacent neighbors of *m*1 in the top table in Fig. 3.

**Figure 4:**
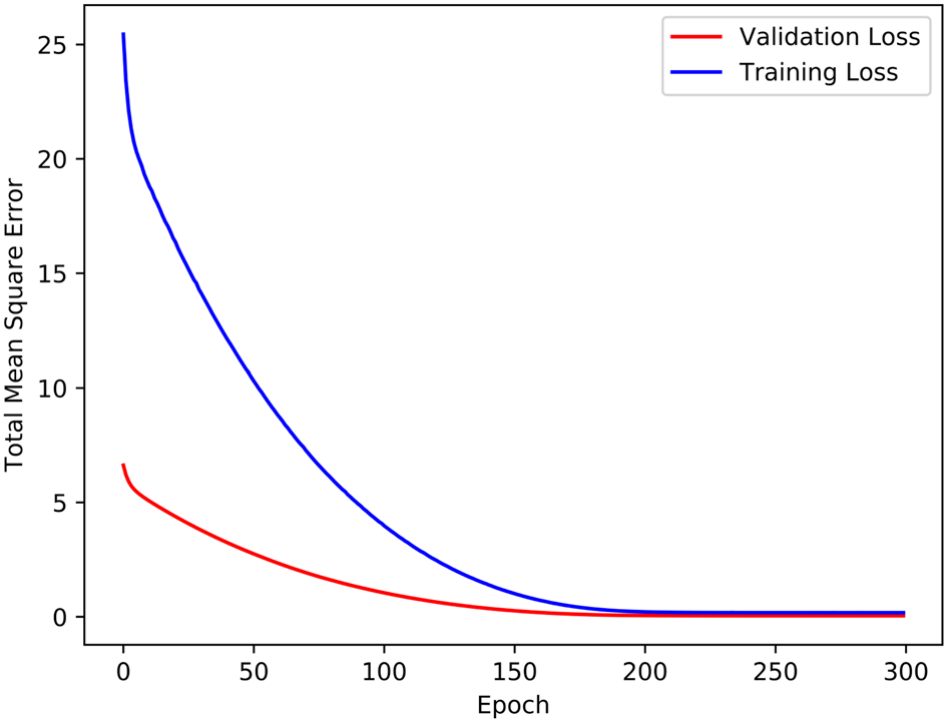
The training and validation error recorded while training the neural layer used to estimate missing BG data points.

We train a one-layer network with eight inputs, the eight adjacent readings of the missing data point, and a single output to estimate all available readings in the patient’s time series given their eight adjacent neighbors. Furthermore, we exclusively use readings whose eight neighbors are known; readings for which one or more adjacent neighbors are missing will not be used for training. Figure 3 further demonstrates that after the ML model has been trained, the adjacent neighbors of *m*1 and *m*2 are fed to the model and the estimates for *m*1 and *m*2 are attained and filled in. To verify the effectiveness of our ML-based estimation method for missing data points, we trained a single neural layer on 80% of all the patients’ BG readings, chosen at random, and used the remaining 20% for validation. The training and validation losses observed are shown in Fig. 4. Throughout the training process, the best Mean Squared Error (MSE) achieved on the validation set was 0.25. Furthermore, we observe that both the training and validation losses follow a trend of exponential decrease, indicating that the proposed ML data estimation method is effective at learning the general pattern of a patient’s BG trends and can thus be used to estimate any missing BG readings for the same patient.

#### Class labeling and data preparation

HbA1c is a continuous measure ranging from $5\%$ (excellent) to $14\%$ (dangerous) average BG measures. However, predicting the exact HbA1c value is extremely challenging due to the dynamic nature of BG fluctuations. Thus, we divide the HbA1c scale into two sets of classes/ranges, transforming the prediction into a classification task. We propose two different class/range splits. The first set of classes consists of four HbA1c ranges indicating good (HbA1c$\le$7.5%), medium (7.5%<HbA1c$\le$9%), poor (9%<HbA1c$\le$12.5%), and uncontrolled (HbA1c>12.5%) levels of diabetes management. The second set of classes consists of six HbA1c ranges indicating normal (HbA1c$\le$6.5%), good (6.5%<HbA1c$\le$7.5%), average (7.5%<HbA1c$\le$8.25%), medium (8.25%<HbA1c$\le$9%), poor (9%<HbA1c$\le$10.5%), and uncontrolled (HbA1c>10.5%) levels of diabetes management. All the class divisions were established based on guidance from a prominent endocrinologist at Sidra Medicine who has expertise in the treatment of diabetes in the pediatric population.

**Table 2:** Split of 200 patients into four (C1–C4) classes based on their true/clinically tested HbA1c test score.

Class	Control level	HbA1c range (%)	Total subjects
C1	Good	HbA1c$\le$7.5	58
C2	Medium	7.5<HbA1c$\le$9	56
C3	Poor	9<HbA1c$\le$12.5	51
C4	Uncontrolled	HbA1c>12.5	35

**Table 3:** Split of 200 patients into six (S1–S6) classes based on their true/clinically tested HbA1c test score.

Class	Control level	HbA1c range (%)	Total subjects
S1	Normal	HbA1c$\le$6.5	31
S2	Good	6.5<HbA1c$\le$7.5	27
S3	Average	7.5<HbA1c$\le$8.25	31
S4	Medium	8.25<HbA1c$\le$9	25
S5	Poor	9<HbA1c$\le$10.5	41
S6	Uncontrolled	HbA1c>10.5	45

Our dataset consists of 200 pediatric participants and Tables 2 and 3 show the number of subjects falling in each class category for the four-class and the six-class division set, respectively, based on their clinically tested HbA1c score. Naturally, the six-class division approach provides a tighter HbA1c prediction range compared to the four-class approach, however, the six-class classification task will be more challenging compared to the four-class method.

## Proposed methodology

**Figure 5:**
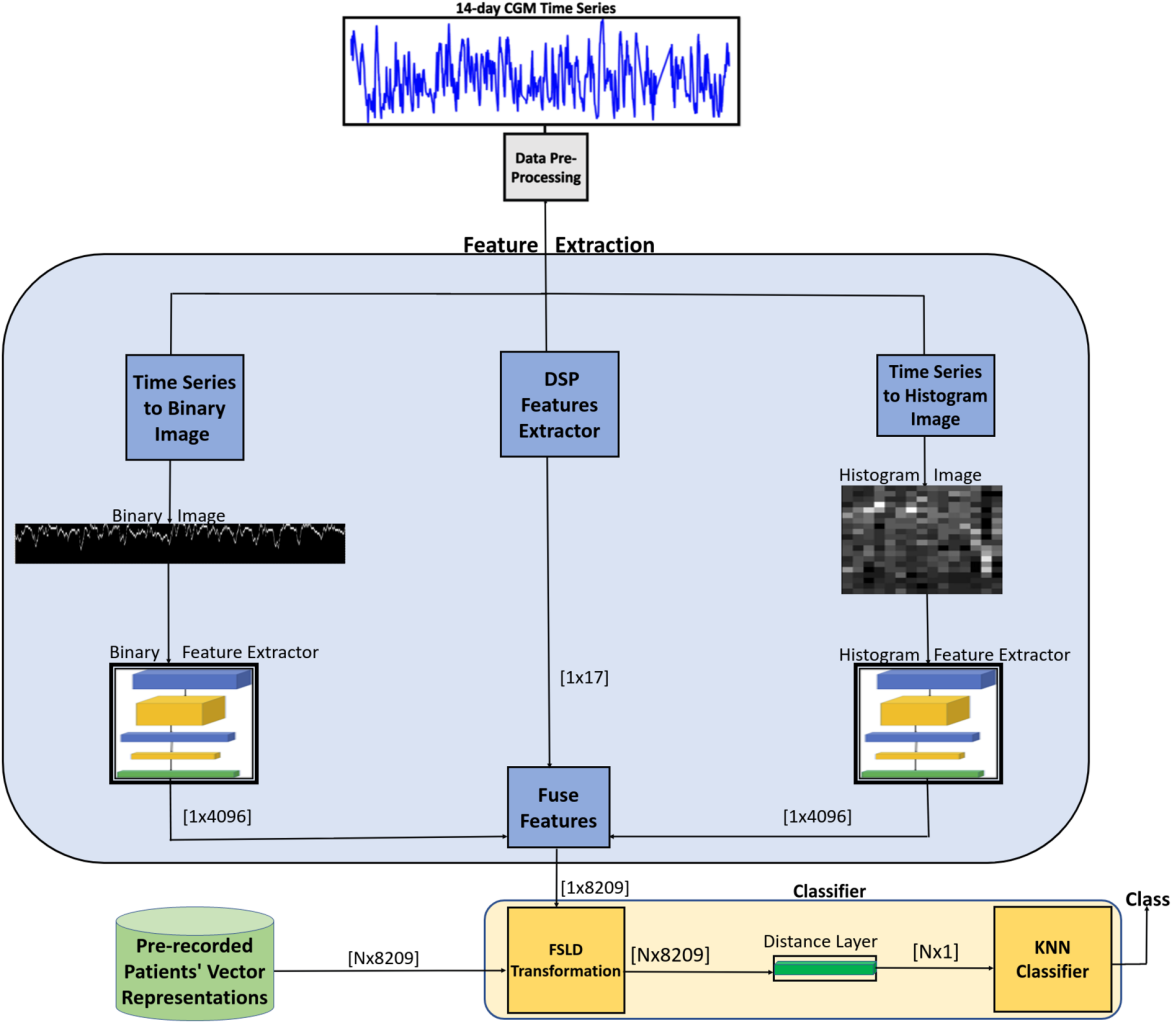
Model architecture for few-shot learning-based feature extraction and fusion for HbA1c prediction.

This section details each stage of the proposed HbA1c prediction model. The proposed HbA1c system architecture is shown in Fig. 5. The first stage of the architecture feeds 14 days of CGM data into a pre-processing unit aimed at filling in missing data from the series, as detailed in Section “Missing data estimation”. In the next step, the CGM time-series data enters a three-channel feature extraction unit. In two of the channels, the CGM time series is first transformed into spatial images which are then fed into CNN-based feature extractor networks. In the third channel, DSP-inspired features are extracted directly from the CGM time series. The features from the three channels are then integrated into the feature fusion unit, which concatenates/combines the three feature vectors. Subsequently, the fused feature vector enters a classification stage, where it first undergoes a novel feature transformation procedure. Finally, the HbA1c class prediction is obtained through a KNN method.

### DSP-based feature extraction

In the DSP feature extraction channel shown in Fig. 5, various signal processing algorithms are implemented to extract pertinent features from the CGM time series. Each DSP technique is designed to derive unique insight from the time series, making connections and correlations that would aid in the HbA1c prediction task.

#### Glucose metabolising capacity

Glucose Metabolising Capacity (GMC) is a measure of a person’s reaction to food consumption^27^. We derive a set of GMC features by adapting the Fractional Derivative (FD) method in^28^. The $k^{th}$ order FD of a function *g*(*x*) is defined as follows:

1 $$\begin{aligned} \small g^{k}(x) = \lim _{h \rightarrow 0} \frac{g(x) - kg(x-h)+\frac{k(k-1)}{2}g(x-2h)+...}{h^k}, \end{aligned}$$

where *k* is the derivative order of function *g*(*x*). For our purposes, Eq. (1) is simplified by only taking the first two terms of the numerator and dividing by the time difference such that

2 $$\begin{aligned} g^{k}(x) = \frac{g(x+h) - kg(x)}{h^k} \end{aligned}$$

The $i-th$ term of the $k^{th}$ order FD of a CGM time series is given by

3 $$\begin{aligned} GMC^{k}_i = \frac{BG_i-kBG_{i+1}}{(t_i-t_j)^k} \end{aligned}$$

where $BG_i$ and $BG_{i+1}$ are consecutive BG readings and $t_i-t_j$ is the time between the readings.

#### Time range features

The time spent within a particular range of BG values is indicative of how well a person with diabetes is managing their disorder. Thus, such measures are correlated with BG control and HbA1c. Time In Range (TIR) measures the proportion of the time that a person’s BG is within some range. TIR measures have been found to have a high correlation with HbA1c^29^. In this study, we extract TIR measures for the ranges of $[70-180~ \text {mg/dL}]$, $[180-250~ \text {mg/dL}]$, $[250-300~ \text {mg/dL}]$, and $[300-350~ \text {mg/dL}]$. These measures are computed as follows

4 $$\begin{aligned} \mathrm {TIR_{70-180}}= & {} \frac{C(70 \le x_i \le 180)}{N}, \end{aligned}$$

5 $$\begin{aligned} \mathrm {TIR_{180-250}}= & {} \frac{C(180 \le x_i \le 250)}{N}, \end{aligned}$$

6 $$\begin{aligned} \mathrm {TIR_{250-300}}= & {} \frac{C(250 \le x_i \le 300)}{N}, \end{aligned}$$

7 $$\begin{aligned} \mathrm {TIR_{300-350}}= & {} \frac{C(300 \le x_i \le 350)}{N}, \end{aligned}$$

where $C(BG_1 \le x_i \le BG_2)$ represents the number of readings that fall in the range $[BG_1, BG_2]$ and *N* is the total number of readings.

#### Coefficient of variability

Coefficient of Variability (CV) is the ratio of the standard deviation, $\sigma$, to the mean, $\bar{x}$, of the CGM time series, and it measures the extent of variability in the BG readings with respect to the mean. The CV feature is computed as follows

8 $$\begin{aligned} CV = \frac{\sigma }{\bar{x}}. \end{aligned}$$

#### Power spectral density

**Figure 6:**
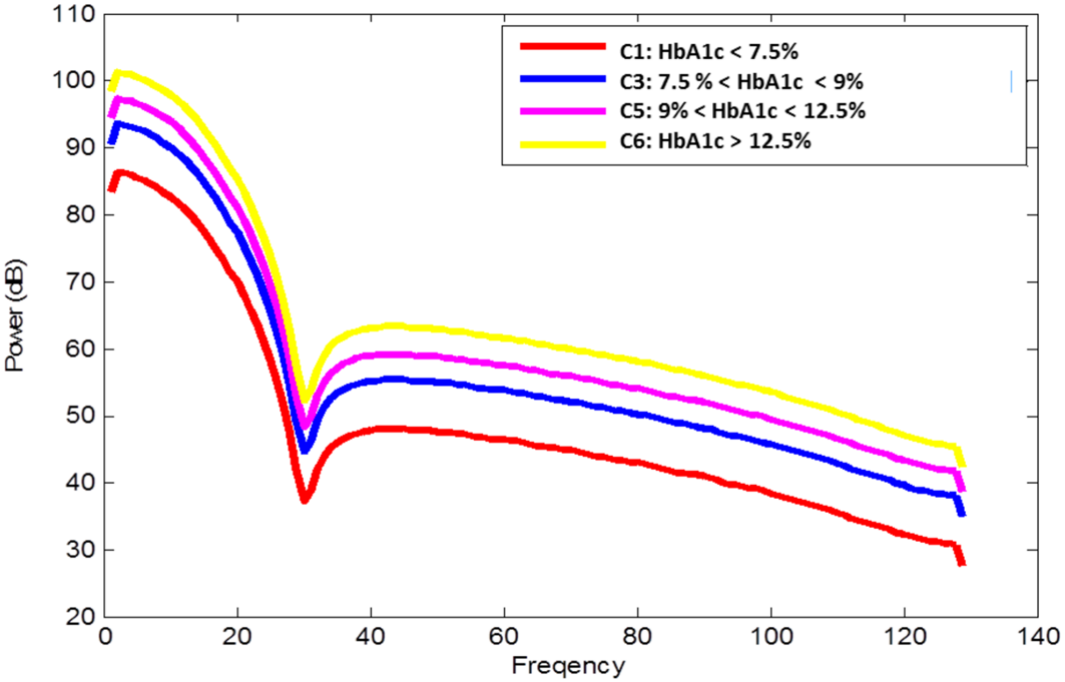
The PSD features comparison among six HbA1c classes.

The Power Spectral Density (PSD) is the power distribution of a signal into its frequency components. Welche’s method^30^ is used to estimate the spectral density at different frequencies. The power in each frequency is added to the DSP-inspired vector by converting a time series signal into its frequency domain components. The PSD is derived using the below formula

9 $$\begin{aligned} S_{xx}(\omega ) = \lim _{T \rightarrow \infty } E [|\hat{x}(\omega )|^2] \end{aligned}$$

where $E[|\hat{x}(\omega )|]$ is the expected value of the signal *x*(*t*) in the frequency domain for some frequency $\omega$. The average spectral densities of the six HbA1c classes are shown below in Fig. 6. As can be seen, certain frequency bands show better separability between the HbA1c class ranges and are ideal for use as features to learn the relationship between CGM and HbA1c.

#### High blood glucose index

High Blood Glucose Index (HBGI) provides a continuous indicator for the risk of hyperglycemia, a condition where the level of sugar in the blood is too elevated, which not only elevates the HbA1c levels but can be life-threatening. The HBGI is computed as follows

10 $$\begin{aligned} HBGI = \frac{\sum _{k=1}^{n} rl(x_k)}{n} \end{aligned}$$

where $x_k$ is the $k-th$ BG reading in the CGM time series *x*, *n* is the total number of BG readings, and *rl*(*x*) is defined as

11 $$\begin{aligned} rl(x) = \left\{ \begin{array}{lr} f(x), &{} \text {if } f(x) < 0\\ 0, &{} Otherwise \end{array} \right\} \end{aligned}$$

where

12 $$\begin{aligned} f(x) = 10 (1.509 (ln(x)^{1.084} - 5.381))^2. \end{aligned}$$

#### Wavelet decomposition

The Discrete Wavelet Transform (DWT), or Wavelet Decomposition (WD), decomposes a signal into a set of basis functions, called wavelets, and has been extensively used for healthcare applications due to their effectiveness in analyzing non-stationary data^31^. Features extracted from the WD technique are called wavelet coefficients. We incorporate Haar WD techniques for feature extraction from CGM data. Specifically, we use the Haar basis coefficients^32^ to obtain three sets of coefficients. Three wavelet decomposition signals, $WD_1$, $WD_2$, and $WD_3$, are obtained and the power of each signal, $P_{WD_{1}}$, $P_{WD_{2}}$, and $P_{WD_{3}}$, is used as a feature.

In addition to the above features, we found that HbA1c levels are highly correlated with BG readings at specific times of the day, namely at 9:00 AM, 10:00 AM, 8:00 PM, and 12:00 AM (midnight). In total, 17 features (listed below) are utilized in the prediction model for their correlation with HbA1c.

- $$GMC^1$$
- $$GMC^{1.5}$$
- $$GMC^2$$
- $$TIR_{300-350}$$
- $$TIR_{70-180}$$
- $$TIR_{250-300}$$
- $$TIR_{180-250}$$
- *CV*
- *PSD*
- *LGBI*
- $$P_{WD_{1}}$$
- $$P_{WD_{2}}$$
- $$P_{WD_{3}}$$
- $$BG_{10AM}$$
- $$BG_{8PM}$$
- $$BG_{9AM}$$
- $$BG_{12AM}$$

### Time-series to binary image transformation

**Figure 7:**
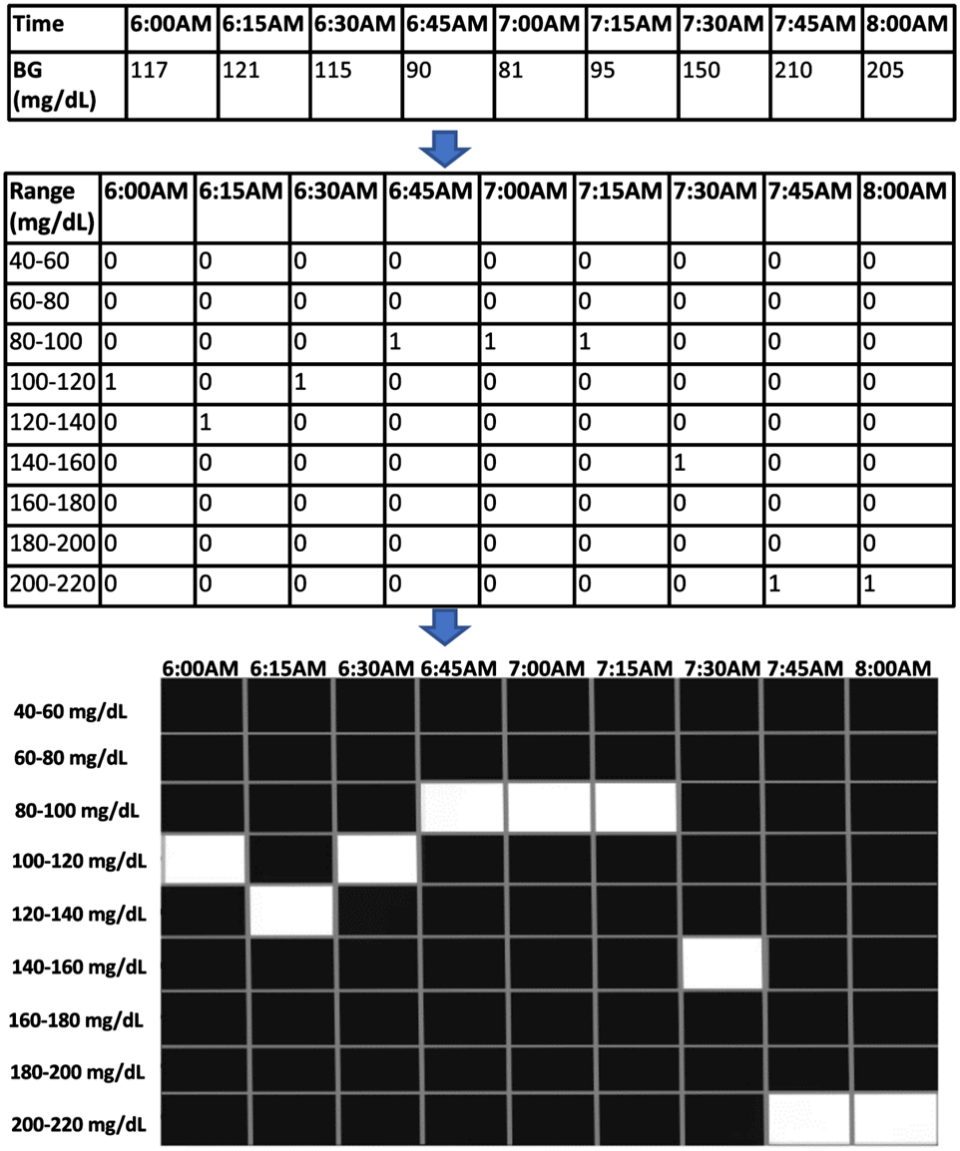
Illustration of the transformation of CGM time-series data into a binary image.

To exploit the benefits of DL models, we develop a data transformation method designed to transform CGM time-series data into binary spatial images. Let ${\textbf {x}}$ be a vector representing 14 days’ worth of CGM data where ${\textbf {x}}$ has *L* BG data points. To obtain a binary image representation of ${\textbf {x}}$, the BG range of 40–500 mg/dL is divided into 20 unit intervals ([40–60 mg/dL), [60–80 mg/dL), and so on) to obtain a total of 24 intervals. A $24 \times L$ binary image is first initialized by setting all pixels to zero intensity (black). The first value of ${\textbf {x}}$ is then mapped to the BG interval it falls within. The pixel encoding its place value is then set to an intensity of 1 (white). In general, if the value of the $i-$th reading of the time series falls within the $j-$th BG interval, then pixel (*i*, *j*) is set to 1. This way, we obtain a binary image where the sequence of white pixels closely resembles the pattern followed by the BG readings in the CGM time series, but the blood glucose interval mapping reduces the dimension of the image. Figure 7 depicts the transformation process described above on a short segment of CGM data for illustrative purposes.

**Figure 8:**
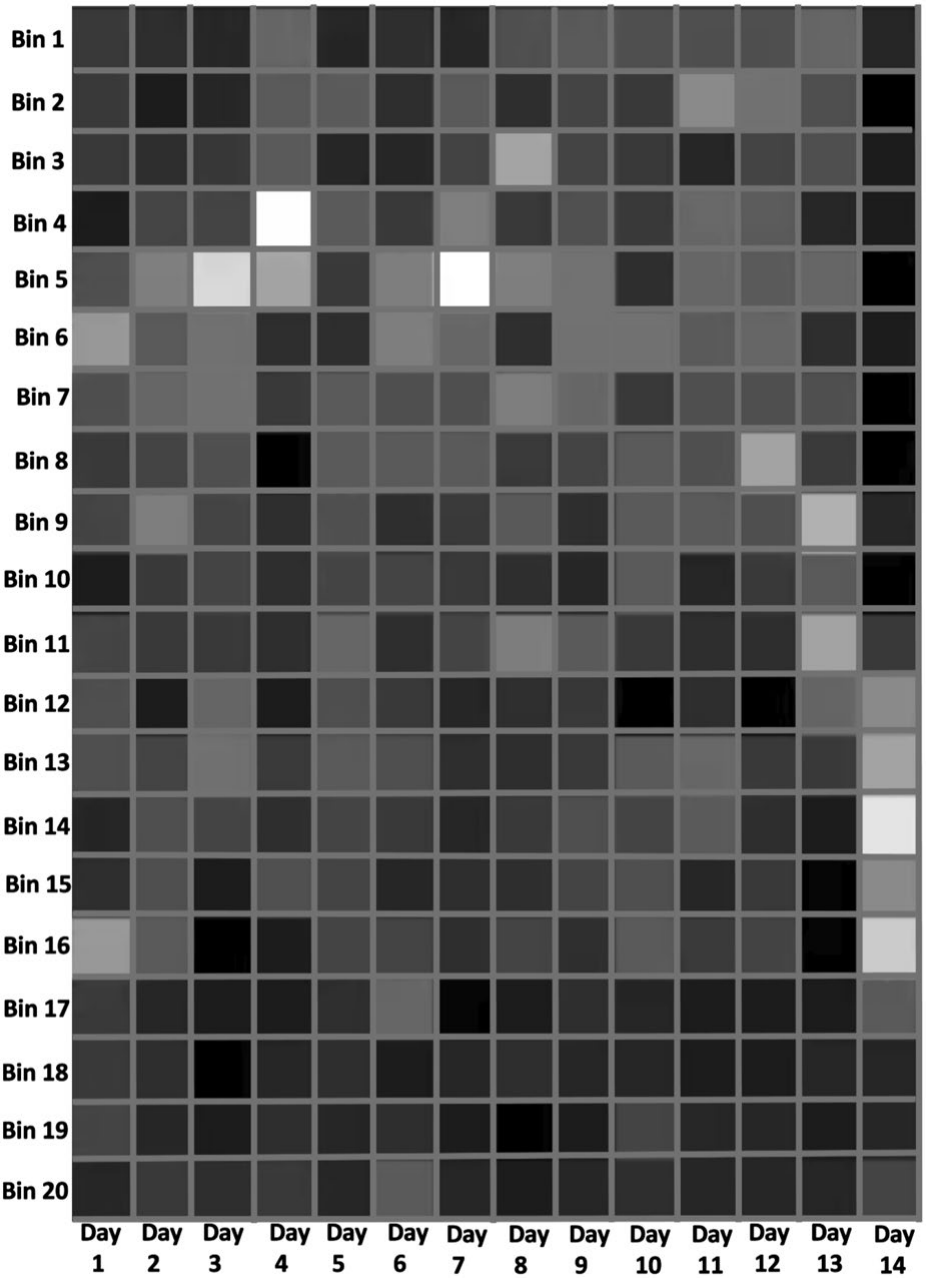
A histogram image that reflects the frequency-distribution of each of 14 days of CGM data.

### Time-series to histogram image transformation

To be able to gain deeper insight from the CGM time-series data, we extract the histogram information in the form of a spatial image. A histogram is a graphical representation of the frequency distribution of a variable of interest. In our case, we use a histogram to describe the frequency distribution of the BG readings on each day of monitoring. We first divide the BG values of each day’s readings into 20 equally-sized intervals, called bins. Assuming that $x_{min}$ and $x_{max}$ are the minimum and maximum BG readings, respectively, in a particular day, this would mean that the 20 intervals into which the BG readings for that day will be divided are $[x_{min}+ni, x_{min}+n(i+1))$ for each $i \in [20]$ where $n=\frac{x_{max} - x_{min}}{20}$. For each of the 14 days, we count the number of times (frequency) the measured BG value falls within each of the 20 bins.

The frequency values for each day are represented by the vector ${\textbf {h}}_{i}$, where ${\textbf {h}}_{i}$ is a $20 \times 1$ column vector and $i\epsilon \left\{ 1,..., 14 \right\}$ indexes the day. The histogram matrix, *H*, of dimension $20 \times 14$, is constructed by concatenating the histogram vector for all 14-days. The matrix is then normalized by the maximum value in *H*. The resulting matrix can be viewed as a grayscale image where each component of the matrix represents the pixel intensity value of a $24 \times 14$ image. Figure 8 depicts a sample of a 14-day CGM histogram image. Brighter pixels indicate longer time spent in that particular BG range.

### Few shot learning-based feature extraction for 2D spatial images

The robustness of deep learning models stems from their intricate architectures, but for these models to perform well, they need to be trained on large amounts of data. However, there are many scenarios where access to large amounts of data is not practical. As we are utilizing 14 days of CGM data to predict the HbA1c class 2.5 months in advance, and we only have 60 or 90 days of CGM data, the amount of training images per HbA1c class is very limited. FSL is a deep learning approach that is designed to classify images based only on a few learning examples^33^. In our proposed system architecture, we utilize the FSL model as a feature extractor (not as a classifier) which allows us to realize the learning capabilities of the FSL model using only a few training images.

**Figure 9:**
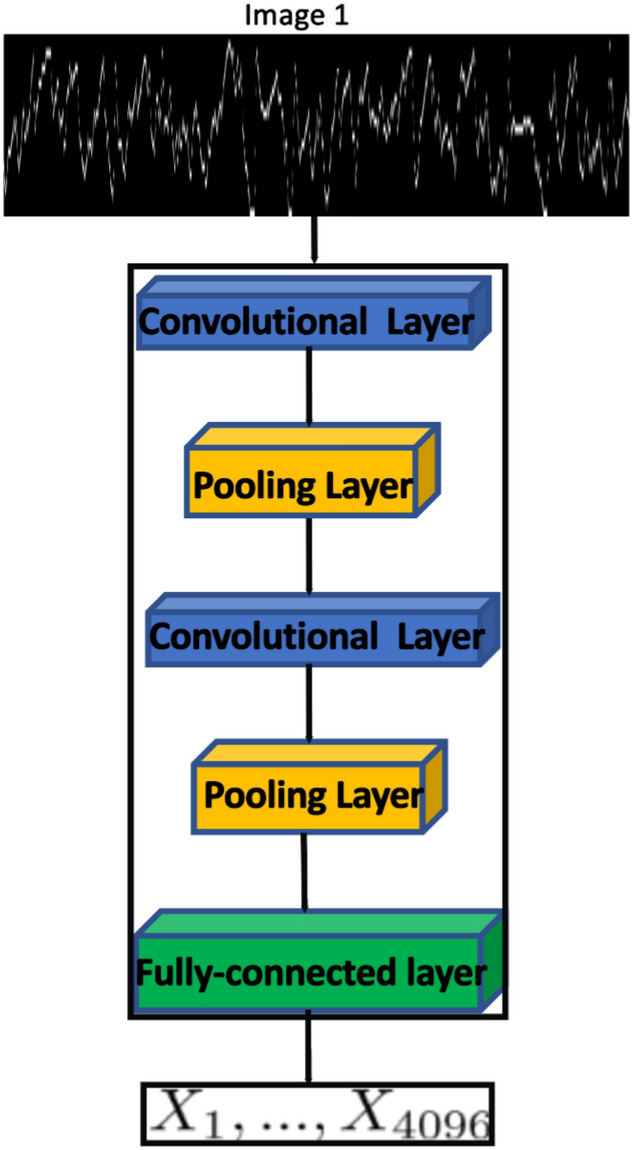
The feature extractor takes an image as input and produces a feature vector of a specified size. In this work, we specify the size of binary and histogram feature vectors to be 4096 features.

**Figure 10:**
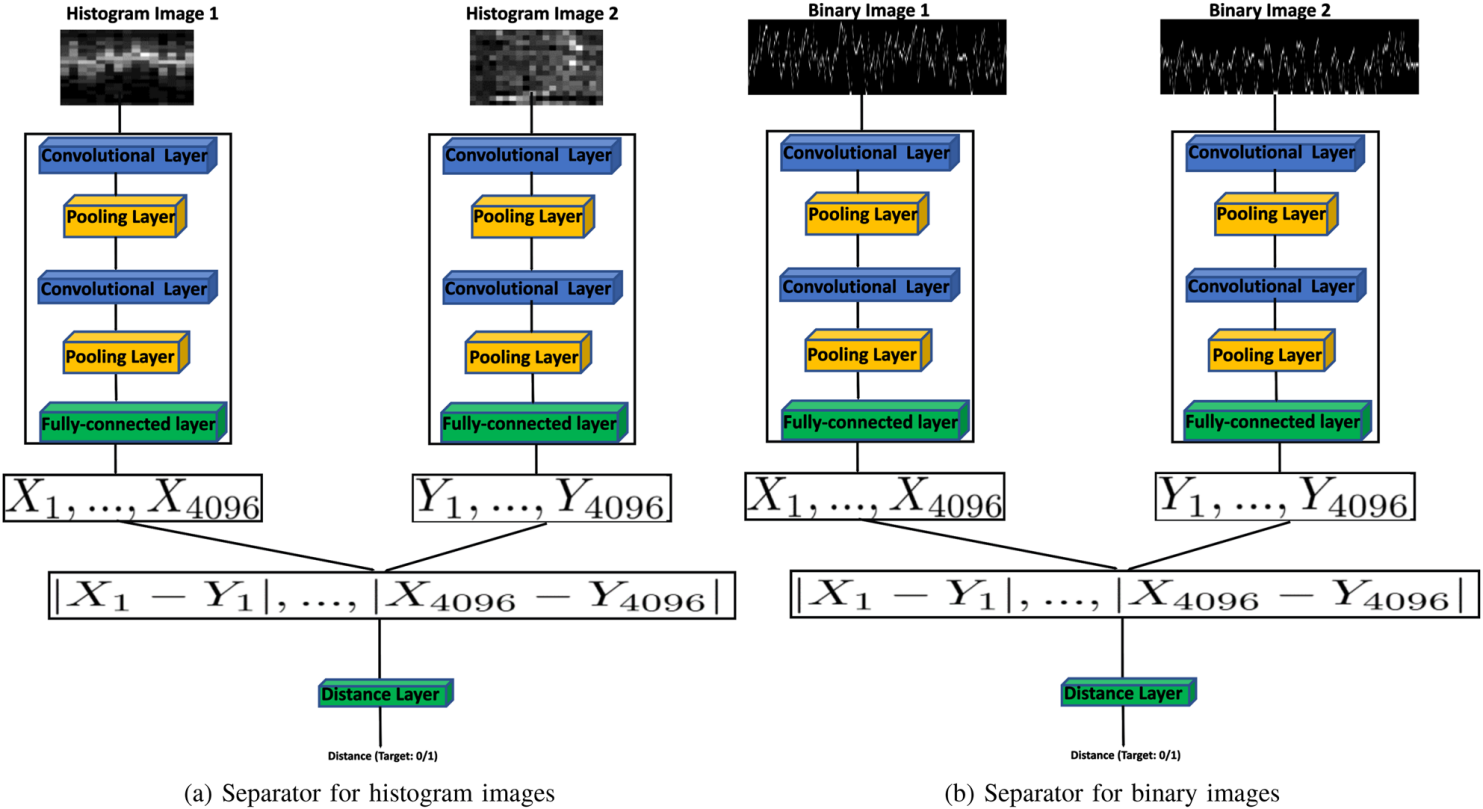
The binary and histogram image separators are trained to separate images representing the CGM time series of patients of different HbA1c classes.

Every generated binary and histogram image is assigned a class label that matches the HbA1C class of the patient whose CGM time series was used to generate that binary or histogram image. For each of the two image representations (binary or histogram), we use FSL to train a CNN-based architecture with two identical CNNs to separate the images based on their classes. This architecture, which we call a “separator”, is shown in Fig. 10. The separator unit takes as input two binary or histogram images and is trained on a target output of zero if the two images belong to the same class and a target output of 1 if the two images belong to different classes. After training, the 4-layer CNN network inside each separator, shown in Fig.  9, is used as a feature extractor. High-level feature vectors of size $[4096 \times 1]$ are obtained from this feature extractor. Our methodology relies on the assumption that the distance, in Euclidean space, between feature vectors obtained from images of the same class will be small relative to the distance between feature vectors obtained from images of different classes. This assumption is reasonable since similar vectors are more likely to achieve the target output of zero for images of the same class.

### Feature fusion

The two image-encoding feature vectors, as well as the DSP-inspired hand-crafted feature vector, are all concatenated to form a single feature vector derived from the CGM time series. To further illustrate the fusion stage, let the feature vector extracted from the binary image representation of the time series be denoted as *B*, the feature vector extracted from the histogram image representation be denoted by *H*, and the statistical feature vector be denoted by

13 $$\begin{aligned} B = \begin{bmatrix} b_{1} \\ \vdots \\ b_{4096} \end{bmatrix} H = \begin{bmatrix} h_{1} \\ \vdots \\ h_{4096} \end{bmatrix} D = \begin{bmatrix} d_{1} \\ \vdots \\ d_{17} \end{bmatrix} \end{aligned}$$

These feature vectors are vertically concatenated to form the consolidated vector, *I*, which is given by

14 $$\begin{aligned} I = \begin{bmatrix} b_{1} \\ \vdots \\ b_{4096}\\ h_{1} \\ \vdots \\ h_{4096}\\ d_1\\ \vdots \\ d_{17} \end{bmatrix} \end{aligned}$$

Note that *I* is an 8209-element vector that constitutes a high-level representation of the class of the time series for which the vector is computed.

### Feature transformation and classification

Recall that the separators of binary and histogram images, shown in Fig. 10, were trained to return a value *close* to 0 for pairs of images that belong to the same class and return a value *close* to 1 for pairs of images that belong to different classes. Consequently, we expect that the Euclidean distance between the feature vectors of images belonging to the same class will be smaller than that between the feature vectors of images belonging to different classes. Additionally, the DSP-based features are selected such that the DSP feature vectors of time series of the same class will be similar, and thus the Euclidean distance between DSP feature vectors of time series that belong to the same class will be smaller than the Euclidean distance between DSP feature vectors of time-series that belong to different classes. Consequently, the Euclidean distance between the fused feature vectors *I* can be reliably used as a class-similarity metric between CGM time series.

To elaborate, consider two time series, $TS_1$, and $TS_2$, that belong to class $C_i$. Since the two time series are from the same class, we expect that: (1) the Euclidean distance between their binary image-based feature vectors, $B_1$ and $B_2$, will be relatively small, (2) the Euclidean distance between their histogram image-based feature vectors, $H_1$ and $H_2$, will be relatively small, and (3) the Euclidean distance between their DSP-based feature vectors, $D_1$ and $D_2$, will be relatively small. Since the overall feature vector of each time-series is a concatenation of the binary image-based, histogram image-based, and DSP-based feature vectors ($I_1 = \{B^T_{1} H^T_{1} D^T_{1}\}^T$ and $I_2 = \{B^T_{2} H^T_{2} D^T_{2}\}^T$), we expect $I_1$ and $I_2$ to be close in Euclidean space. On the other hand, if $TS_1$ and $TS_2$ were from different classes, the Euclidean distance between their respective overall feature vectors $I_1$ and $I_2$ would be larger. Thus, we say that the Euclidean distance between the overall feature vectors of two time series can be used as a class-similarity metric.

Conventionally, the FSL model adopts the absolute distance measure^34^ and in the literature, it was evaluated for alphabet classification using a publicly available onmiglot dataset^34^ and achieved an 88% accuracy for 1-shot learning with convolutional Siamese nets. However, in our work, we propose a novel FSLD metric instead of the absolute difference to quantify the difference between two feature vectors. Specifically, we want to optimize the separation between the different HbA1c classes, thus we design the FSLD metric to amplify the difference between feature vectors of time series that belong to different classes while minimizing the distance between feature vectors of time series that belong to the same class. The proposed FSLD metric has the potential to separate images of different categories effectively. We begin with the assumption that the difference between corresponding elements of feature vectors of time series that belong to the same class is small relative to the difference between corresponding elements of feature vectors of time series that belong to different classes. Namely, for any two feature vectors $I_1 = (i^1_{1}, \dots ,i^{4096}_{1})^T$ and $I_2 = (i^1_{2}, \dots ,i^{4096}_{2})^T$ that were constructed from time-series that belong to the same class, we assume that $|i^j_{1} - i^j_{2}|$ will be relatively small for each $j \in [4096]$. This is a safe assumption since the Euclidean distance between $I_1$ and $I_2$ is presumed to be small if $I_1$ and $I_2$ are constructed from time series that belong to the same class. We compute a “difference vector” $I_d$ from $I_1$ and $I_2$ where $I_d = (|i^1_{1}-i^1_{2}|, \dots ,|i^{4096}_{1}-i^{4096}_{2}|)^T$. Note that $I_d$ reflects the closeness between $I_1$ and $I_2$– elements $i_d \in I_d$ will be relatively small if $I_1$ and $I_2$ are from time-series that belong to the same class and relatively large (close to 1) otherwise. To increase the degree to which $I_d$ reflects class-similarity between $I_1$ and $I_2$, we apply an FSLD transformation to $I_d$, producing an FSLD-transformed vector **J**. Our FSLD transformation amplifies large differences through an exponential function and floors elements below a certain threshold *a* to 0. This is done as follows:

15 $$\begin{aligned} I_1= & {} \begin{bmatrix} i_{1}^1 \\ \vdots \\ i_{1}^{4096} \end{bmatrix} I_2 = \begin{bmatrix} i_{2}^1 \\ \vdots \\ i_{2}^{4096} \end{bmatrix} \end{aligned}$$

16 $$\begin{aligned} I_d= & {} \begin{bmatrix} |i_{1}^1 - i_{2}^1| \\ \vdots \\ |i_{1}^{4096} - i_{2}^{4096}| \end{bmatrix} \end{aligned}$$

17 $$\begin{aligned} {{\textbf {J}}}= & {} \begin{bmatrix} FSLD(i_{1}^1, i_{2}^1) \\ \vdots \\ FSLD(i_{1}^{4096}, i_{2}^{4096}) \end{bmatrix} \end{aligned}$$

where

18 $$\begin{aligned} FSLD(i_1,i_2) = \left\{ \begin{array}{ll} 0 &{} |i_1-i_2| \le a \\ \frac{|i_1-i_2|}{max(|i_1|, |i_2|)} &{} Otherwise \end{array} \right. \end{aligned}$$

The FSLD-transformed vector **J** is a vector that encodes the “clossness” between the classes of the CGM time series represented by $I_1$ and $I_2$. In order to learn the “clossness” encoded by *I*, we use supervised learning to train a one-layer distance model whose input is FSLD-transformed vectors, such as **J**, and whose output is a single value between 0 and 1. To train this distance model, we generate all possible vectors **J** from all possible vectors $I_1$ and $I_2$, which are extracted from all possible pairs of time series $TS_1$ and $TS_2$. During training, each input vector **J** is associated with a target output of 1 if $TS_1$ and $TS_2$ belong to the same class. Otherwise, the target output of the model is set to 0. Note that this one-layer distance model produces a pseudo-distance metric between the two, time series used to compose its input. This one-layer distance model is likely to output small values (close to 0) for vectors **J** that were composed of two, time series that belong to the same class. Conversely, The one-layer distance model is likely to output large values (close to 1) for vectors **J** that were composed of two, time series that belong to different classes.

To classify a vector representation of a CGM time series, we adopt a K-Nearest Neighbor (KNN) approach. Our choice of the KNN as the classifier is based on its superior performance efficiency compared to other machine learning models that were also tested, including a support vector machine (SVM), a decision tree, and a shallow neural network (NN). First, we compute the distances between the vector of interest and the vectors of all other time series whose classes are known using the FSLD metric described above. Our prediction of the class of the given time series is the class that appears most frequently in the set of the *K* “closest” time series. A value of $K=10$ was found to be optimal for the KNN model through experimentation. Note that the task at hand is a multi-class classification task. This means that a tie case of having an equal number of nearest neighbors belonging to different classes is possible whether *K* is even or odd; If $K = 9$, for instance, we may encounter a scenario where 3 of the 9 nearest neighbors belong to class *C*1, 3 of the 9 nearest neighbors belong to class *C*2, 3 of the 9 nearest neighbors belong to class *C*3, and none of the 9 nearest neighbors belong to class *C*4. To resolve a tie case between two or more classes, we choose a random class from the set of tied classes as output to the KNN module.

## Performance evaluation and discussion

In this section, we evaluate the performance of the proposed CGM-based HbA1c prediction architecture by training and testing it on the attained dataset. The data from 80% of the patients was used to train the architecture, and the data from the remaining 20% of patients was used for testing. In addition, we benchmark the proposed classifier against a publicly available image dataset to signify the robustness of the proposed FSL-based feature extraction and FSLD discriminator.

### Performance analysis of long-term HbA1c prediction

We evaluate the performance based on the accuracy of the prediction over a set of patient data. The accuracy is calculated as follows

19 $$\begin{aligned} \textrm{Accuracy} = \frac{D_C}{D_T}* 100 \end{aligned}$$

where $D_C$ is the number of correctly classified instances and $D_T$ represents the total number of instances.

In our proposed approach, we integrate features from the following: (i) binary images transformed from the CGM data, (ii) histogram images generated from the CGM data, and (iii) DSP-based features extracted from CGM data. However, to evaluate the effectiveness of each of the feature extraction approaches, we additionally evaluate the proposed prediction model using each feature extraction channel independently (i.e., using a binary image-based model independently, a histogram image-based model, and a DSP feature-based model). We trained and tested our model for the four-class HbA1c scheme and for the six-class HbA1c scheme. Table 4 shows the results of our model for the four-class separation scheme. We note that the utilization/integration of all the developed feature channels proves to achieve the best performance, achieving a test accuracy of $89.25\%$. The inference time for all 7 combinations of features was very close, with the inference time being 1.13ms ± 0.1ms. These calculations were conducted on a system equipped with an AMD Ryzen 9 3950x 16-core processor and an NVIDIA GeForce RTX 2080Ti GPU.

**Table 4:** Performance analysis of the four-class separation HbA1c prediction for binary image input, histogram image input, and DSP-based features as an input, and all combinations of the three representations.

Binary histogram DSP			Average five-fold accuracy (%)	Standard deviation
		$\checkmark$	85.04	2.81
	$\checkmark$		85.19	2.30
	$\checkmark$	$\checkmark$	88.32	4.04
$\checkmark$			88.74	1.17
$\checkmark$		$\checkmark$	89.03	2.29
$\checkmark$	$\checkmark$		87.37	2.57
$\checkmark$	$\checkmark$	$\checkmark$	89.25	1.16

The developed model has also been evaluated when the HbA1c range is divided into six classes/intervals, thus providing a tighter prediction of the true HbA1c level (clinical HbA1c). As seen from Table 5, the integration of the three-channel features again attains the best performance compared to using each feature extraction channel independently for prediction. We notice a $2.41\%$ drop in the average accuracy compared to the 4-class HbA1c division, which is expected as it becomes more challenging to predict accurately 2.5 months in advance the HbA1c level when the class range is stringent. These outcomes highlight the challenges to be faced if we attempt to set up our problem as a regression problem and try to predict the exact continuous HbA1c value instead of predicting in which class/interval the predicted HbA1c will fall. The average inference time, on the other hand, did not change significantly for the 6-class and came out at 1.12ms $\pm 0.01$ms for all combinations of features on the same PC used to train our model on the 4-class case mentioned above.

**Table 5:** Performance analysis of the six-class separation HbA1c prediction for binary image input, histogram image input, and DSP-based features as an input, and all combinations of the three representations.

Binary histogram DSP			Average five-fold accuracy (%)	Standard deviation
		$\checkmark$	82.38	1.6
	$\checkmark$		81.17	2.74
	$\checkmark$	$\checkmark$	82.80	1.14
$\checkmark$			83.68	0.69
$\checkmark$		$\checkmark$	82.20	1.51
$\checkmark$	$\checkmark$		80.12	1.45
$\checkmark$	$\checkmark$	$\checkmark$	86.84	1.41

As highlighted in the introduction, to the best of our knowledge, there has been no work, other than our initial study^35^, that targets the futuristic prediction of HbA1c levels of pediatric patients using short-term CGM data. Thus, we do not have the opportunity to benchmark our proposed model against other similar work in the literature. However, we did attain a performance enhancement in terms of prediction accuracy when we compare the proposed approach in this paper to our work in^35^, which was based on a multi-stage machine learning architecture.

### Benchmarking on other datasets/applications

**Figure 11:**
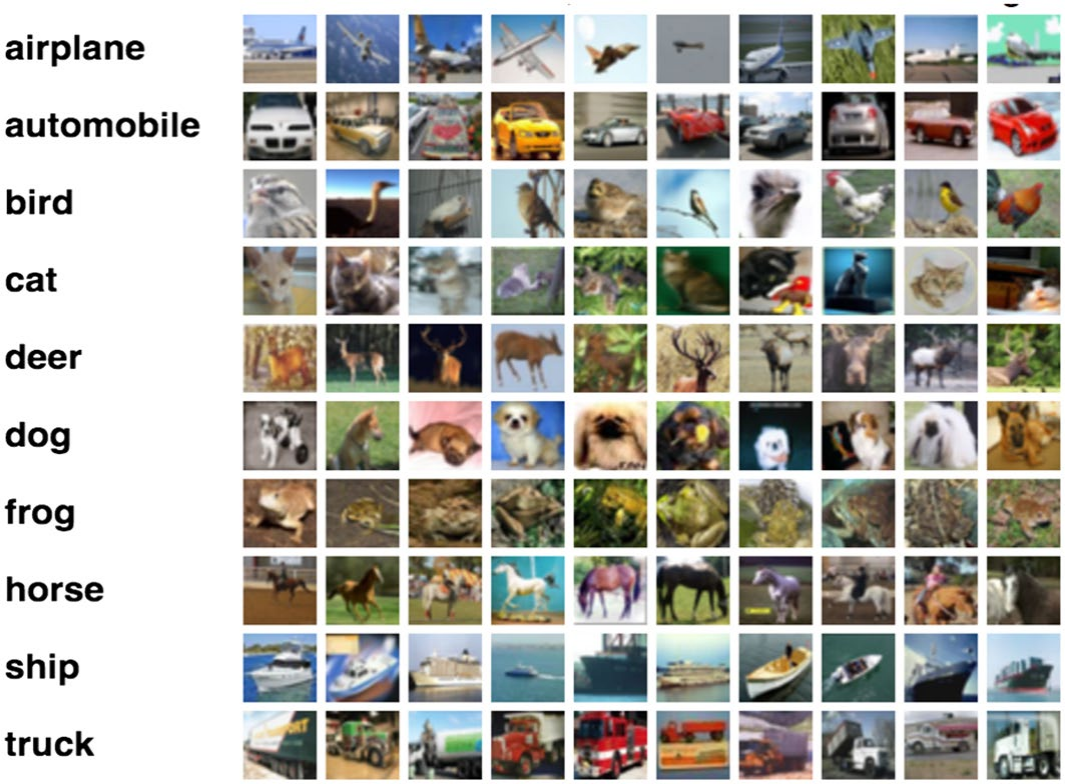
The ten classes of CIFAR10 dataset^36^.

The developed framework can be generalized to other image-based classification tasks for real-world applications where available data is limited. Here, we evaluate the performance of our proposed FSL-based feature extraction and FSLD approach using a publicly available benchmark dataset. Specifically, we have tested our framework on the CIFAR10 dataset^36^, which comprises 50,000 training images and 10,000 testing images of ten classes, as shown in Fig. 11. Table 6 outlines the performance of the 10-class image classification task when adopting our proposed FSL-based architecture FSL-based feature extraction coupled with FSLD on the CIFAR10 dataset, and the results are noteworthy.

The proposed model achieved the highest test accuracy of 94.89% during differentiating images from the airplane category vs. all other image categories. The model obtained an overall average accuracy of 93.20% when tested on its ability to discriminate all image classes.

**Table 6:** Evaluation of the proposed FSL-based model on the publicly available CIFAR10 dataset.

Classification task	Train accuracy (%)	Test accuracy (%)
Airplane versus rest	96.5%	94.89
Automobile versus rest	97.41	92.75
Bird versus rest	95.55	94.10
Cat versus rest	95.12	93
Deer versus rest	93.95	90.43
Dog versus rest	95.67	94.35
Frog versus rest	96.75	93.88
Horse versus rest	92.32	91.90
Ship versus rest	95.67	93.11
Truck versus rest	96.15	93.54
Overall	95.51	93.20

To further evaluate the robustness of our proposed architecture, we compare the performance of the classification task on the 10-image classification of the CIFAR10 dataset using several well-known image classification algorithms. The results obtained, shown in Table 7, show that our proposed approach performs comparable to the state-of-the-art models in the literature while only using a fraction (5%) of the original dataset to implement our FSL-based feature extraction approach. In addition, the training of our model on smaller datasets is significantly faster compared to conventional CNN-based models, which must be trained on larger datasets to attain similar performance. Last but not least, our proposed approach does not require the utilization of powerful workstations with high GPU capabilities. We are able to run our model using a machine with a CPU (Intel Core i5, 2.50GHz Processor, 8GHz RAM). This feature is extremely important when designing lightweight models that can be hosted on wearable devices. In our context, HbA1c prediction, the ultimate aim is to be able to embed the developed algorithm in smart devices (smartwatches, smartphones, etc.) so that people living with diabetes can (i) continuously *evaluate* their predicted HbA1c values based on their current behavior/lifestyle and CGM trends, (ii) *assess* how their current behavior and BG measures impact their long-term HbA1c, and (iii) make the necessary *changes* to enable better control of their disorder.

**Table 7:** The comparison of the proposed FSL-derived model with the state-of-the-art on CIFAR10.

Study	Method	Test accuracy (%)
^37^	PCANet Baseline	78.7
^38^	Bayesian Optimization	90.5
^39^	ReNet: RNN	87.7
^40^	VGG-19	94.71
Proposed architecture	FSL-derived Model	93.20

## Conclusion

Diabetes is a disorder that requires continuous monitoring and evaluation. BG tracking is a standard monitoring technique, and now, with the development of wearable BG measuring sensors, the monitoring task has been simplified. Yet, the management of diabetes continues to be a reactive one, where the treatment plan is adjusted after HbA1c measures indicate poor control of BG. In this work, we proposed a long-term HbA1c prediction algorithm based on short-term CGM data. The concept of futuristic prediction of clinical HbA1c measures using current CGM data is revolutionary despite its potential benefits and impact on the management of diabetes in both pediatric and adult patients.

In the proposed algorithm, we transform CGM sensor time-series data into binary and histogram images to further improve HbA1C prediction. The generated images are passed through a CNN framework for FSL-based feature extraction. The extracted features are fused with DSP-based features and then processed using a proposed FSLD metric, which quantifies the distances between images. A thresholding hyperplane was established to distinguish the distances of images from the same class to the distances of images from different classes. Finally, a KNN model was implemented and test images were assigned to the class with majority vote counting. Performance analysis shows very good prediction accuracy for both the four-class and the six-class HbA1c division approaches when utilizing the lightweight, FSL-derived algorithm, motivating its incorporation in smart devices for *proactive* management of diabetes.

## Data Availability

The data that support the findings of this study are available from Sidra Medicine but restrictions apply to the availability of these data, which were used under license for the current study, and so are not publicly available. Data are however available from the authors upon reasonable request and with permission of the Institutional Review Board (IRB) of Sidra Medicine.
